# Vascular complications and bleeding after balloon aortic valvuloplasty performed with or without heparin: HEPAVALVE randomized study

**DOI:** 10.1016/j.ijcha.2021.100951

**Published:** 2022-01-18

**Authors:** Mariama Akodad, Jessica Labour, Erika Nogue, Delphine Delseny, Jean-Christophe Macia, Richard Gervasoni, Benoit Lattuca, Nicolas Nagot, François Roubille, Guillaume Cayla, Florence Leclercq

**Affiliations:** a Department of Cardiology, CHU Montpellier, Univ Montpellier, Montpellier, France; b PhyMedExp, Uni Montpellier, INSERM, CNRS, France; c Clinical Research and Epidemiology Unit, CHU Montpellier, Univ Montpellier, Montpellier, France; d Department of Cardiology, CHU Nimes, Univ Montpellier, Nimes, France

**Keywords:** Balloon aortic valvuloplasty, Vascular and bleeding complications, Aortic stenosis, Percutaneous interventions

## Abstract

**Background:**

Vascular and bleeding events remain the main complications after balloon aortic valvuloplasty (BAV). While BAV is usually performed with per procedural heparin injection, BAV performed without heparin may reduce hemorrhagic events. We aimed to determine whether vascular and bleeding complications may be reduced with BAV performed without heparin.

**Methods:**

This randomized, double-blind, placebo-controlled study was conducted from January 2013 to September 2016. Patients were randomly assigned to placebo or intravenous unfractionated heparin (UH) 50 IU/kg bolus during the procedure. The primary endpoint included major vascular, bleeding and ischemic complications (stroke, transient ischemic attack, myocardial infarction) according to VARC-2 criteria.

**Results:**

Among 89 randomized patients, 82 completed the study (n = 39 in the UH group and n = 43 in the placebo group). At baseline, diabetes, sex male and renal failure were more frequent in the UH group and peripheral artery disease was more frequent in the placebo group. The primary endpoint was achieved in 7 patients (8.5%), 1 in the placebo group (2.3%) versus 6 in the UH group (15.4%). After adjustment on diabetes, sex, renal failure, peripheral artery disease, percutaneous closure device and chronic obstructive pulmonary disease, UH utilization was associated with a significant risk of major vascular, bleeding and ischemic complications (primary endpoint) (_adj_OR: 11.9; 95%CI: 1.2–117.2; p = 0.03). Hospitalization length was lower in the placebo group compared to the UH group (p = 0.03).

**Conclusions:**

BAV without per procedural UH was associated with a reduction of major VC and bleeding events without increasing the ischemic risk and with a shorter hospitalization length.

## Highlights

• Vascular and bleeding events remain frequent after balloon aortic valvuloplasty.
• Balloon aortic valvuloplasty without per procedural heparin is associated with a reduction of major VC and bleeding events.
• Balloon aortic valvuloplasty without per procedural heparin is not associated with an increased risk of ischemic complications.

## 1 Introduction

Balloon aortic valvuloplasty (BAV) was developed in 1985 by Professor Cribier for patients with aortic stenosis (AS) contraindicated to surgical aortic valve replacement [1], [2]. The hopes of this technique gradually faded due to early restenosis and lack of improvement in mortality [2], [3], [4]. A new technique, the transcatheter aortic valve replacement (TAVR), was developed to overcome these issues relegating BAV to a palliative treatment, according to guidelines [5], [6]. Nevertheless, BAV remains useful as a rescue therapy in patients with cardiogenic shock eligible for definitive therapy, as a bridge to TAVR or surgery, in ambiguous cases as a therapeutic test, to allow emergency surgery or as a palliative therapy [7], [8], [9]. Despite improvements in BAV technique with reduced sheath size, vascular complications (VC) remain the main issue after BAV, whereas serious complications as aortic rupture, severe aortic regurgitations (AR) or death represent <3% of events [5], [10], [11], [12], [13], [14]. Indeed, bleeding and VC were reported as occurring in 6–17.6% of patients after BAV [5], [10], [11], [12], [13], [14]. Thus, as this technique is usually performed in frail patients with comorbidities, reduction of these complications is critical. Unfractionated heparin (UH) is almost systematically used during BAV but this strategy is totally empirical and raises the question both of its utility to reduce ischemic complications and its impact on VC and bleeding complications. Our team previously conducted a non-randomized study including 162 patients comparing heparin and non-heparin BAV procedures with an increased risk of bleeding without lowering ischemic events in the heparin BAV group [15].

The objective of this study was to confirm by a randomized study whether the procedure of BAV without administration of per procedural heparin may decrease the risk of bleeding complications without increasing the risk of ischemic events compared to the usual procedure using heparin.

## 2 Methods

### 2.1 Study design

The HEPAVALVE study was a randomized, double-blind trial conducted and sponsored by Montpellier University Hospital, France. All patients with severe AS, confirmed by transthoracic echocardiography (TTE) (mean aortic gradient >40 mmHg and/or aortic valve area <1 cm^2^), with indication of BAV according to European guidelines (6) were included from January 2013 to September 2016 in Montpellier University Hospital, France. Patients considered as ineligible for the study included those with inaccessible femoral approach, hemodynamic failure, AR >grade 2, hemorrhagic disease, known contra-indication to heparin or to local anesthesia, under guardianship and pregnant or breastfeeding women. Administration of a low molecular weight heparin <12 h or of UH < 4 h before the procedure, or vitamin K antagonist (VKA) treatment with international normalized ratio (INR) >1.5 were exclusion criteria.

### 2.2 Data assessment and ethics regulation

The study was conducted by the cardiology team of Montpellier University Hospital with a steering committee comprising the investigators, the methodologist, the representative of the promoter and the biostatistician was set up to direct the study. An independent judgment criteria validation committee, composed of senior cardiologists, blindly evaluated all clinical events based on the medical data received to perform optimal referring. Funding was obtained from Edwards Lifesciences. The study was conducted according to the ethical principles of the 1975 Helsinki Declaration, bioethics laws. The protocol was approved by an independent ethics committee. A written informed consent was obtained from each patient prior to randomization. The study was registered with ClinicalTrials.gov (NCT01823393).

### 2.3 Study treatments and procedure

**Fig. 1:**
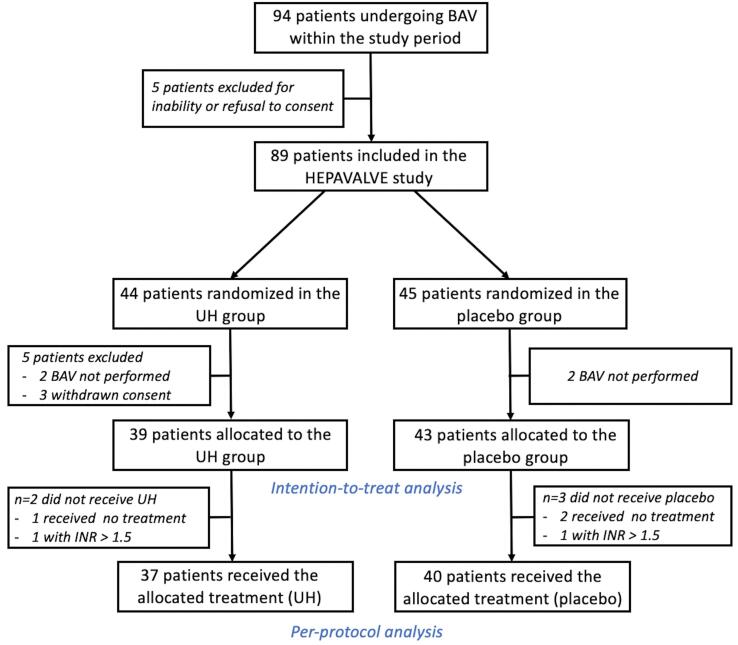
Study flow chart.

Two groups of patients were constituted: control group *(*group UH) who benefited of conventional BAV with UH injection (50 IU/kg) and experimental group *(*group placebo) with injection of placebo (sodium chloride) (Fig. 1). UH (Heparin choay® Sanofi-Aventis France) and placebo (Sodium Chloride PROAMP 0.9% Aguettant laboratory) were prepared immediately after randomization by our institution’s pharmacy and delivered to the unit as soon as possible with identical conditioning to guarantee the blind. BAV procedure was performed by a team of 3 experienced cardiologists, according to a standardized technique using a retrograde femoral approach. According to the result of the randomization, UH (50 IU/ kg) or placebo was administered by intravenous bolus at the beginning of the procedure just after femoral sheath insertion (8 or 9 French).

Coronary angiography was systematically performed before the procedure and coronary angioplasty, if necessary, was performed before BAV, at the latest the day before. The crossing of the aortic valve was performed under fluoroscopic control by 0.034 in. straight guide through Amplatz left 1 or 2. Pressure gradient between the left ventricle and aorta was measured with average and peak to peak gradients. BAV was performed using non-compliant balloons (Tyshak™ balloon catheters, B Braun) with a size selected according to left ventricle outflow track (LVOT) diameter assessed by TTE. To stabilize balloon position through the aortic valve prior to inflation, rapid stimulation of the right ventricle was performed (180–200 bpm) until systolic blood pressure <80 mmHg was reached. BAV result was considered satisfactory by a mean gradient decrease >40% or >20 mmHg. In the opposite case and in the absence of complication, a new BAV was performed with the use of a higher diameter balloon. At the end of the procedure, post-procedural hemodynamic transvalvular gradient was evaluated and the presence or absence of AR was systematically assessed with contrast aortography. The arterial puncture site was closed with a vascular closure device (8-F Angio-Seal TM, St. Jude Medical, St. Paul, MN, US) or with manual compression (with or without the use of a Femoral compression device (Femostop™, Abbott)) according to the operator’s choice.

### 2.4 Endpoints

The primary endpoint was a composite endpoint of major VC, major bleeding and major ischemic complications according to VARC-2 criteria at 1-month follow-up [16].

Major VC included: 1/access site or access related vascular injury (arteriovenous fistulas, pseudoaneurysms, stenosis, dissection, hematoma, percutaneous device failure) requiring unplanned intervention AND/OR leading to death, life-threatening or major bleeding, visceral ischemia or neurological impairment, 2/distal-embolization (non-cerebral) from vascular source requiring surgery or resulting in amputation or irreversible end-organ damage, 3/New ipsilateral lower extremity ischemia. Bleeding was defined as major in case of bleeding ≥BARC 3 [16]. Major ischemic complications included stroke or transient ischemic attack confirmed by cerebral imaging and myocardial infarction (new ischemic symptoms and troponin elevation >15 upper reference limit) [16].

The secondary endpoint included minor VC and minor bleeding and analysis of all VC, bleeding, ischemic complications (any severity) according to VARC-2 criteria [16]. Death, AR >grade 2 and hospitalization length were also assessed.

### 2.5 Randomization and statistical analysis

The randomization was performed by random blocks with 6 and 8 size permutation and a 1:1 ratio between the two arms. Once consent was obtained and inclusion criteria were verified, patient randomization was performed by Ennov Clinical® software, also used for data assessment and management. The retrospective analysis carried out in our department showed that a severe complication (composite outcome) occurred among 25% of patients who received heparin *vs*. 4% of those who did not receive heparin. In order to detect such a difference with 90% power with a 5% alpha risk, we needed to enroll 120 patients. This number was increased to 130 to account for patients lost to follow-up. Patient characteristics were described at baseline for the Per Protocol (PP) and Intention-To-Treat (ITT) populations and treatment groups (UH and placebo groups) with medians and interquartile ranges (P_25_-P_75_) for quantitative variables and frequencies and proportions for categorical variables. The occurrence of endpoints was described between treatment groups. The effect size of treatment was estimated with the odds ratio (OR) and its 95% confidence interval (95%CI) adjusted on covariates clinically different between treatment groups at the inclusion. Firth logistic regression was used when quasi-complete separation problem occurred. The effect of treatment on the hospitalization length was also studied using linear regression with adjustment on pertinent covariates, adjusted regression coefficient (_adj_β) associated to heparin use was reported with its 95% confidence interval (95%CI). Statistical analyzes were implemented using SAS (Enterprise Guide, version 7.13; SAS Institute; Cary, North Carolina, USA).

## 3 Results

### 3.1 Baseline and procedural characteristics

Between January 2013 and September 2016, 89 consecutive patients were randomized (Fig. 1). Indication for BAV was palliative in 33 patients (37.1%), as a bridge for TAVR or surgical aortic valve replacement in 33 patients (37.1%) and before non-cardiac surgery in 23 patients (25.8%) without difference between UH and placebo groups. Among randomized patients, 82 were finally analyzed including 39 (47.6%) UH patients and 43 (52.4%) placebo patients. In the UH group, 2 patients did not receive the allocated treatment *vs.* 3 patients in the placebo group, 77 patients were then considered in the PP population (Fig. 1). In the ITT population, the median age was 86 years (Q_25_–Q_75_: 80–88) with NYHA class 3 or 4 symptoms for 58 patients (76.3%). Both groups were comparable at baseline except for diabetes, sex male, chronic obstructive pulmonary disease and renal failure, more frequent in the UH group, and for peripheral artery disease, more frequent in the placebo group (Table 1). Vascular closure device was used in most patients (89%) but appeared more frequently used in the placebo group (95.2% *vs*. 81.6%) (Table 1).

**Table 1:** Baseline and procedural characteristics of the two treatment groups in ITT and PP populations.

	ITT population n = 82			Per Protocol population n = 77		
	All ITT	UH group n = 39	Placebo group n = 43	All PP	UH group n = 37	Placebo group n = 40
***Patient characteristics***						
Age (Years)*	86 (80–89)	86 (81–88)	85 (77–89)	86 (79–88)	86 (81–88)	83.5 (77–89)
**Male Sex***, n (%)*	37 (45.1)	20 (51.3)	17 (39.5)	34 (44.2)	19 (51.4)	15 (37.5)
BMI (km/m^2^)*	25.6 (22.7–28.7)	25.0 (22.7–26.6)	25.9 (23.4–30.4)	25.4 (22.7–28.0)	24.8 (22.2–26.5)	25.9 (23.4–30.4)
**Hypertension***, n (%)*	50 (61.7)	24 (61.5)	26 (61.9)	49 (64.5)	24 (64.9)	25 (64.1)
**Coronary artery disease**	30 (37.0)	15 (39.5)	15 (34.9)	28 (36.8)	14 (38.9)	14 (35.0)
**Previous stroke***, n (%)*	7 (8.6)	4 (10.5)	3 (6.9)	7 (9.2)	4 (11.1)	3 (7.5)
**Permanent pacemaker***, n (%)*	10 (12.2)	6 (15.4)	4 (9.3)	10 (12.9)	6 (16.2)	4 (10.0)
**COPD***, n (%)*	22 (26.8)	12 (30.8)	10 (23.3)	19 (24.7)	11 (29.7)	8 (20.0)
**Atrial fibrillation***, n (%)*	37 (46.3)	18 (48.7)	19 (44.2)	33 (44.0)	16 (45.7)	17 (42.5)
**Diabetes***, n (%)*	22 (27.2)	15 (39.5)	7 (16.3)	20 (26.3)	13 (36.1)	7 (17.5)
LVEF (%)*	45 (35–50)	43 (35–45)	47.5 (35.5–65)	44 (35–52.5)	43 (35–45)	45 (35–70)
**Peripheral artery disease***, n (%)*	10 (12.4)	3 (7.9)	7 (16.3)	10 (13.2)	3 (8.3)	7 (17.5)
**NYHA ≥ 3***, n (%)*	58 (76.3)	26 (72.2)	32 (80.0)	54 (76.1)	24 (70.6)	30 (81.1)
**Renal failure***, n (%)*	16 (19.5)	10 (25.6)	6 (13.9)	12 (15.6)	8 (21.6)	4 (10.0)
**Hemoglobin < 120 mmHg***, n (%)*	40 (48.8)	23 (59.0)	17 (39.5)	37 (48.1)	21 (56.8)	16 (40.0)

**Antithrombotic regimen***, n (%)*						
None	9 (10.9)	4 (10.3)	5 (11.6)	9 (11.7)	4 (10.8)	5 (12.5)
SAPT	28 (34.2)	14 (35.9)	14 (32.6)	27 (35.1)	14 (37.8)	13 (32.5)
DAPT	14 (17.1)	7 (17.9)	7 (16.3)	14 (18.2)	7 (18.9)	7 (17.5)
Anticoagulant alone	17 (20.7)	7 (17.9)	10 (23.3)	14 (18.2)	5 (13.5)	9 (22.5)
Anticoagulant + SAPT	11 (13.4)	5 (12.8)	6 (13.9)	10 (12.9)	5 (13.5)	5 (12.5)
Anticoagulant + DAPT	3 (3.7)	2 (5.1)	1 (2.3)	3 (3.9)	2 (5.4)	1 (2.5)

***Procedural characteristics***						
**Percutaneous closure device,***n (%)*	71 (88.8)	31 (81.6)	40 (95.2)	68 (88.3)	30 (81.1)	38 (95.0)

**Femoral sheath size (French)***, n (%)*						
*8 French*	74 (93.7)	35 (94.6)	93 (92.9)	72 (93.5)	35 (94.6)	37 (92.5)
*9 French*	4 (5.1)	2 (5.4)	2 (4.8)	4 (5.2)	2 (5.4)	2 (5.0)
Number of inflations*	2 (2–3)	2 (2–3)	2 (2–3)	2 (2–3)	2 (2–3)	2 (2–3)
Balloon size (mm)*	22 (20–22)	22 (22–22)	22 (20–22)	22 (20–22)	22 (22–22)	22 (20–22)
Mean aortic gradient pre-BAV (mmHg)*	42 (30–56)	44 (32–58)	40 (28–56)	42 (30–56)	44 (32–58)	40 (28–56)
Mean aortic gradient post-BAV (mmHg)*	17 (10–28)	20 (10–28)	17 (8–29)	20 (10–28)	20 (10–28)	17 (8–29)

BAV: balloon aortic valvuloplasty; BMI: body mass index; COPD: chronic obstructive pulmonary disease; DAPT: dual antiplatelet therapy; GI: gastro-intestinal, ITT: intention-to-treat; LVEF: left ventricular ejection fraction; NYHA. New York Heart Association; PP: per-protocol; SAPT: simple antiplatelet therapy; UH: unfractionned heparin.

* Quantitative variables are expressed as median (Q_25_ − Q_75_).

### 3.2 Primary endpoint

**Fig. 2:**
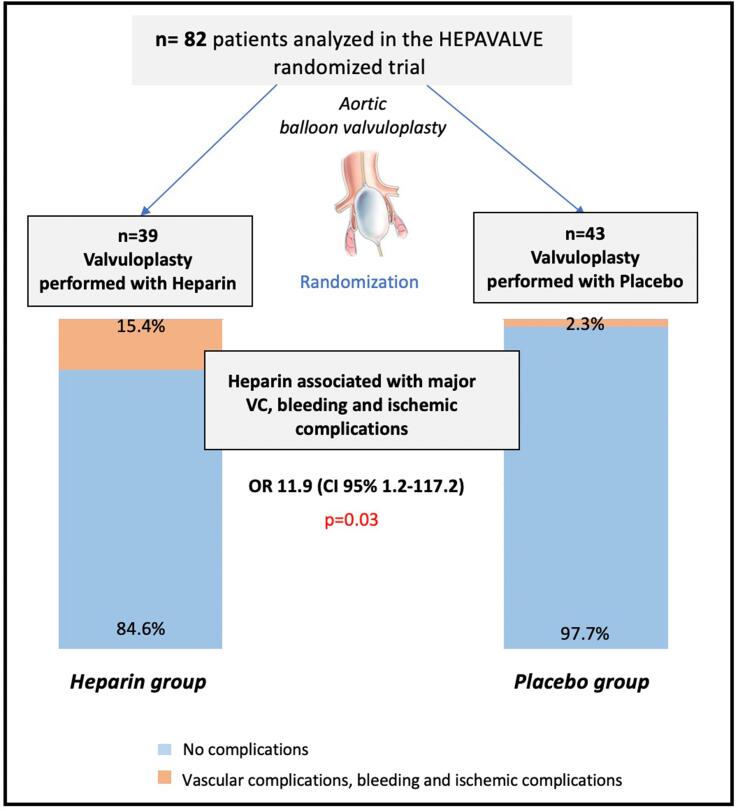
Major VC, bleeding, and ischemic events according to UH and placebo groups (primary endpoint), UH: unfractionated heparin; VC: vascular complication.

In the ITT population, the primary endpoint occurred in 7 patients (8.5%) including 6 (15.4%) complications in the UH group *vs* 1 (2.3%) in the placebo group (Fig. 2). Major VC were the most common adverse events occurring in 4 patients (4.9%) with major bleeding involving 1 patient (1.2%). Major ischemic events including ischemic stroke were observed in 3 patients (3.7%), all in the UH group. After adjustment on diabetes, sex, renal failure, peripheral artery disease, chronic obstructive pulmonary disease and percutaneous closure device, the administration of per procedural heparin appeared significantly associated to the risk of major vascular, bleeding and ischemic complications (primary endpoint) with an adjusted OR of 11.9 [95%CI: 1.2–117.2], p = 0.03 (Table 2). In the PP analysis, the effect of heparin on primary endpoint was consistent to the ITT analysis (supplemental Table 1). Antithrombotic regimen was similar between patients with and without major complications (supplemental Table 2), p = 0.98. Details on antithrombotic regimen for each patient with major complication are provided in supplemental Table 3.

**Table 2:** Endpoints assessment among patients receiving either heparin or placebo for balloon aortic valvuloplasty (Intention-to-treat analysis).

	All ITT N = 82	UH group n = 39	Placebo groupn = 43	_adj_OR [95% CI] *UH vs. Placebo*	p
**Primary endpoint**	**7 (8.5)**	**6 (15.4)**	**1 (2.3)**	**11.9 [1.2**–**117.2]**	**0.03**
Major VC*	4 (4.9)	3 (7.7)	1 (2.3)	6.4 [0.6–76.1]	0.1
Major bleeding*	1 (1.22)	1 (2.6)	0 (0.0)	6.4 [0.5–83.9]	0.2¤
Major ischemic complication**	3 (3.7)	3 (7.7)	0 (0.0)	6.8 [0.6–76.5]	0.1¤
**Secondary endpoint**	**8 (9.8)**	**5 (12.8)**	**3 (7.0)**	**2.0 [0.3**–**14.0]**	**0.5**
Minor VC	1 (1.2)	0 (0.0)	1 (2.3)	0.6 [0.06–5.5]	0.6¤
Minor bleeding**	7 (8.5)	5 (12.8)**	2 (4.7)	3.1 [0.4–26.6]	0.3
**Primary or secondary endpoint**	**14 (17.1)**	**10 (25.6)**	**4 (9.3)**	**4.4 [1.0**–**19.0]**	**0.049**
**Total vascular and bleeding**	**12 (14.6)**	**8 (20.5)**	**4 (9.3)**	**3.4 [0.7**–**16.8]**	**0.1**

ITT: intention-to-treat; VC: vascular complications.

p_adj_: p value of multivariate logistic regression with treatment group as covariate adjusted on diabetes, sex, coronary artery disease, renal failure and percutaneous closure device; ^¤^ p value estimated with Firth logistic regression.

* The same patient presented major VC and major bleeding.

** The same patient presented minor bleeding and major ischemic complication.

### 3.3 Secondary endpoints

Minor VC or minor bleeding occurred in 8 patients (9.8%) without significant difference between groups (_adj_OR: 2.0 [0.3–14.0]; p = 0.5). Total hemorrhagic, vascular or ischemic complications (all severity) appeared less frequent in the placebo group *vs.* the UH group (_adj_OR: 4.4 [1.0–19.0]; p = 0.049). The per protocol analysis was consistent with the intent-to-treat analysis (Table 2 and supplemental Table 1).

There was no thrombus formation in the catheter during or after the procedure in both groups.

Overall, 1 patient (2.6%) died during follow-up in the UH group. Death was related to cardiogenic shock associated with severe left ventricular dysfunction. AR > grade 2 was observed in 2 patients (5.1%) in the UH group. Hospitalization length, with a mean duration of 5 ± 4 days in the UH group *vs*. 3 ± 2 days in the placebo group, appeared significantly higher in the UH group (_adj_β: 2.04; 95%CI: 0.2–3.8]; p = 0.03) in the ITT population as in the PP population (_adj_β: 2.01; 95%CI: 0.2–3.9]; p = 0.03) after adjustment on same covariates used for primary endpoint analyses.

## 4 Discussion

We assessed for the first time in a randomized study the impact of BAV performed without heparin with 3 main findings:

1. In patients undergoing BAV, heparin administration was associated with a significant increased risk of major complications including VC, bleeding and ischemic complication
2. BAV performed without heparin did not increase ischemic events
3. Hospitalization length was higher in the heparin group

### 4.1 VC and bleeding after BAV

VC and bleeding after BAV remain common, between 5% and 11% in registries (10, 11, 15). Indeed, patients with severe AS are particularly prone to VC related to age, polyvascular disease or acquired coagulopathy [17]. In addition, post-procedure immobilization can be altered by individual parameters such as confusion or cardiac decompensation. Major VC rate of the whole population in our study was 4.9%, comparable with others studies. In contrast, severe hemorrhagic complications appeared to be less frequent (1.2%) than in the literature suggesting a link with the discontinuation of heparin in our study [10], [11], [12], [13], [18]. We observed a significant increase in combined major vascular, bleeding and ischemic complications with the administration of heparin after adjustment on potential confounding factors. The results of this trial are consistent with our previous non-randomized study on 162 patients undergoing BAV suggesting a significant reduction in bleeding complications without increased ischemic risk in the absence of heparin during the procedure [15]. The utilization of percutaneous closure devices in the majority of patients may also explain our relative low rates of VC and bleeding. Finally, heparin may increase bleeding and VC in patients undergoing BAV, especially in patients with increased hemorrhagic risk, particularly frequent in this frail population with severe AS.

### 4.2 Ischemic events and BAV

In previous studies, BAV was associated with 1% of ischemic complications as opposed to vascular and hemorrhagic complications which were more common reaching 5–11% [10], [11], [15]. Here, ischemic events compared favorably with these results with only 3 ischemic events (stroke) in our study, all in the UH group. A bolus of UH between 2500 and 7500 IU is routinely administered at the beginning of BAV without any recommendations [10], [11], [19]. Indeed, in all interventional procedures, heparin is used to prevent thromboembolic complications. The passage of the wire through atheromatous arteries, aortic arch as well as through the aortic valve can be traumatic and may lead to systemic embolic complications. In the TAVR era, several studies focused on the nature of these emboli, which appears to be mainly composed of calcic material [20], [21]. Kahlert et al. showed that silent embolic events at the brain level concerned nearly 86% of patients undergoing TAVR and appeared mainly at the time of prosthesis implantation and not during BAV, this being confirmed by transcranial Doppler performed during the procedure [20]. Our findings are in favor of the absence of benefit of UH on potential calcic emboli during BAV, especially in this short procedure as the risk of thromboembolism may increase with procedure length.

### 4.3 Hospitalization length

In our study, hospitalization length was higher in the UH group. This may be explained by the higher rate of hemorrhagic and vascular complications in this group of patients. Indeed, complications are well-known associated with increased morbidity and need for surgery leading to increase hospitalization length [10], [22]. Indeed, in a recent study including 17,672 patients undergoing percutaneous interventions using large diameter devices, the authors highlighted a higher mortality (OR 2.7; 95% CI, 2.3–3.2; p < 0.001), longer hospitalization length of stay (OR 2.1; 95% CI, 2.1–2.2; p < 0.001) and higher health care costs (OR 1.6; 95% CI, 1.5–1.6; p < 0.001) in patients with bleeding complications [22]. Thus, BAV without UH may decrease hospitalization length and costs by decreasing the incidence of hemorrhagic complications.

### 4.4 Limitations

First, the monocentric nature of the study may induce bias. Secondarily, despite randomization, groups of patients were not totally homogeneous, including twice as many diabetic patients in the UH group, although diabetes did not emerge as a predictor of complication and all analysis were adjusted on confounding factors. Third, activated clotting time was not measured in the study in the UH group, to maintain the study blind, not allowing to assess heparin efficacy. Finally, the sample size was lower than expected at the beginning of the study but allowed to demonstrate significant impact of heparin on the primary endpoint.

## 5 Conclusion

In conclusion, this randomized trial shows that avoiding heparin may reduce major vascular bleeding and ischemic complications in patient undergoing BAV. This strategy could contribute to decrease both duration of hospitalization and costs after BAV.

## Funding

This study was funded by the University Hospital of Montpellier, avenue du doyen Giraud, 34295 Montpellier (France) by an unrestricted grant from Edwards Lifesciences, route de l’Etraz 70, 1260 Nyon (Switzerland).

## Disclosures

M. Akodad received research grants from Edwards Lifescience, Medtronic and Fédération Française de cardiologie.

B. Lattuca received research grants from ACTION Study group, Biotronik, Boston Scientific, Daiichi-Sankyo, Fédération Française de Cardiologie and Institute of CardioMetabolism and Nutrition; consultant fees from Daiichi-Sankyo and Eli Lilly; and lecture fees from AstraZeneca and Novartis.

G Cayla received research grants/consultant fees/lectures fees from Amgen, AstraZeneca, Bayer, Boehringer-Ingelheim, Boston, Biotronik, Bristol-Myers Squibb, Daiichi-Sankyo, Eli-Lilly, Europa, Fédération Française de Cardiologie, Fondation Cœur & Recherche, Medtronic, MSD, Pfizer, Sanofi-Aventis

F. Leclercq received research grants from Edwards, Medtronic, Boehringer; consultant fees from Boehringer; and lecture fees from Astra Zeneca and Bayer.

## Declaration of Competing Interest

The authors declare that they have no known competing financial interests or personal relationships that could have appeared to influence the work reported in this paper.

## References

[1] Cribier A., Saoudi N., Berland J., Savin T., Rocha P., Letac B. (1986). Percutaneous transluminal valvuloplasty of acquired aortic stenosis in elderly patients: an alternative to valve replacement. Lancet.

[2] Lieberman E.B., Bashore T.M., Hermiller J.B., Wilson J.S., Pieper K.S., Keeler G.P., Pierce C.H., Kisslo K.B., Harrison J.K., Davidson C.J. (1995). Balloon aortic valvuloplasty in adults: failure of procedure to improve long-term survival. J. Am. Coll. Cardiol..

[3] Otto C.M., Mickel M.C., Kennedy J.W., Alderman E.L., Bashore T.M., Block P.C., Brinker J.A., Diver D., Ferguson J., Holmes D.R. (1994). Three-year outcome after balloon aortic valvuloplasty. Insights into prognosis of valvular aortic stenosis. Circulation.

[4] Letac B., Cribier A., Eltchaninoff H., Koning R., Derumeaux G. (1991). Evaluation of restenosis after balloon dilatation in adult aortic stenosis by repeat catheterization. Am. Heart J..

[5] Eltchaninoff H., Durand E., Borz B., Furuta A., Bejar K., Canville A., Farhat A., Fraccaro C., Godin M., Tron C., Sakhuja R., Cribier A. (2014). Balloon aortic valvuloplasty in the era of transcatheter aortic valve replacement: acute and long-term outcomes. Am. Heart J..

[6] Baumgartner H., Falk V., Bax J.J., De Bonis M., Hamm C., Holm P.J., Iung B., Lancellotti P., Lansac E., Rodriguez Muñoz D., Rosenhek R., Sjögren J., Tornos Mas P., Vahanian A., Walther T., Wendler O., Windecker S., Zamorano J.L., Roffi M., Alfieri O., Agewall S., Ahlsson A., Barbato E., Bueno H., Collet J.-P., Coman I.M., Czerny M., Delgado V., Fitzsimons D., Folliguet T., Gaemperli O., Habib G., Harringer W., Haude M., Hindricks G., Katus H.A., Knuuti J., Kolh P., Leclercq C., McDonagh T.A., Piepoli M.F., Pierard L.A., Ponikowski P., Rosano G.M.C., Ruschitzka F., Shlyakhto E., Simpson I.A., Sousa-Uva M., Stepinska J., Tarantini G., Tchétché D., Aboyans V., Windecker S., Aboyans V., Agewall S., Barbato E., Bueno H., Coca A., Collet J.-P., Coman I.M., Dean V., Delgado V., Fitzsimons D., Gaemperli O., Hindricks G., Iung B., Jüni P., Katus H.A., Knuuti J., Lancellotti P., Leclercq C., McDonagh T., Piepoli M.F., Ponikowski P., Richter D.J., Roffi M., Shlyakhto E., Simpson I.A., Zamorano J.L., Kzhdryan H.K., Mascherbauer J., Samadov F., Shumavets V., Camp G.V., Lončar D., Lovric D., Georgiou G.M., Linhartova K., Ihlemann N., Abdelhamid M., Pern T., Turpeinen A., Srbinovska-Kostovska E., Cohen A., Bakhutashvili Z., Ince H., Vavuranakis M., Temesvári A., Gudnason T., Mylotte D., Kuperstein R., Indolfi C., Pya Y., Bajraktari G., Kerimkulova A., Rudzitis A., Mizariene V., Lebrun F., Demarco D.C., Oukerraj L., Bouma B.J., Steigen T.K., Komar M., De Moura Branco L.M., Popescu B.A., Uspenskiy V., Foscoli M., Jovovic L., Simkova I., Bunc M., de Prada J.A.V., Stagmo M., Kaufmann B.A., Mahdhaoui A., Bozkurt E., Nesukay E., Brecker S.J.D. (2017). 2017 ESC/EACTS Guidelines for the management of valvular heart disease. Eur. Heart J..

[7] Ussia G.P., Capodanno D., Barbanti M. (2010). Balloon aortic valvuloplasty for severe aortic stenosis as a bridge to high-risk transcatheter aortic valve implantation. J. Invasive Cardiol..

[8] Sandhu K., Krishnamoorthy S., Afif A., Nolan J., Gunning M.G. (2017). Balloon aortic valvuloplasty in contemporary practice. J. Interv. Cardiol..

[9] Moreno P.R., Jang I.-K., Newell J.B., Block P.C., Palacios I.F. (1994). The role of percutaneous aortic balloon valvuloplasty in patients with cardiogenic shock and critical aortic stenosis. J. Am. Coll. Cardiol..

[10] Ben-Dor I., Pichard A.D., Satler L.F., Goldstein S.A., Syed A.I., Gaglia M.A., Weissman G., Maluenda G., Gonzalez M.A., Wakabayashi K., Collins S.D., Torguson R., Okubagzi P., Xue Z., Kent K.M., Lindsay J., Waksman R. (2010). Complications and outcome of balloon aortic valvuloplasty in high-risk or inoperable patients. JACC Cardiovasc. Interv..

[11] Percutaneous balloon aortic valvuloplasty. Acute and 30-day follow-up results in 674 patients from the NHLBI Balloon Valvuloplasty Registry. Circulation 84 (1991) 2383–2397. 10.1161/01.cir.84.6.2383 1959194

[12] Don C.W., Witzke C., Cubeddu R.J. (2010). Comparison of procedural and in-hospital outcomes of percutaneous balloon aortic valvuloplasty in patients >80 years versus patients < or =80 years. Am. J. Cardiol..

[13] Kapadia S.R., Goel S.S., Yuksel U., Agarwal S., Pettersson G., Svensson L.G., Smedira N.G., Whitlow P.L., Lytle B.W., Tuzcu E.M. (2010). Lessons learned from balloon aortic valvuloplasty experience from the pre-transcatheter aortic valve implantation era. J. Interv. Cardiol..

[14] Saia F., Marrozzini C., Ciuca C., Guastaroba P., Taglieri N., Palmerini T., Bordoni B., Moretti C., Dall’Ara G., Branzi A., Marzocchi A. (2013). Emerging indications, in-hospital and long-term outcome of balloon aortic valvuloplasty in the transcatheter aortic valve implantation era. EuroIntervention.

[15] Leclercq F., Delseny D., Nogue E. (2014). Decrease of vascular and bleeding complications after balloon aortic valvuloplasty performed without heparin. J. Invasive Cardiol..

[16] Kappetein A.P., Head S.J., Généreux P., Piazza N., van Mieghem N.M., Blackstone E.H., Brott T.G., Cohen D.J., Cutlip D.E., van Es G.-A., Hahn R.T., Kirtane A.J., Krucoff M.W., Kodali S., Mack M.J., Mehran R., Rodés-Cabau J., Vranckx P., Webb J.G., Windecker S., Serruys P.W., Leon M.B. (2013). Valve Academic Research Consortium-2. Updated standardized endpoint definitions for transcatheter aortic valve implantation: the Valve Academic Research Consortium-2 consensus document. J. Thorac. Cardiovasc. Surg..

[17] Massyn M.W., Khan S.A. (2009). Heyde syndrome: a common diagnosis in older patients with severe aortic stenosis. Age Ageing.

[18] Hara H., Pedersen W.R., Ladich E., Mooney M., Virmani R., Nakamura M., Feldman T., Schwartz R.S. (2007). Percutaneous balloon aortic valvuloplasty revisited: time for a renaissance?. Circulation.

[19] McKay R.G. (1991). The Mansfield Scientific Aortic Valvuloplasty Registry: Overview of acute hemodynamic results and procedural complications. J. Am. Coll. Cardiol..

[20] Kahlert P., Al-Rashid F., Döttger P., Mori K., Plicht B., Wendt D., Bergmann L., Kottenberg E., Schlamann M., Mummel P., Holle D., Thielmann M., Jakob H.G., Konorza T., Heusch G., Erbel R., Eggebrecht H. (2012). Cerebral embolization during transcatheter aortic valve implantation: a transcranial Doppler study. Circulation.

[21] Omran H., Schmidt H., Hackenbroch M., Illien S., Bernhardt P., von der Recke G., Fimmers R., Flacke S., Layer G., Pohl C., Lüderitz B., Schild H., Sommer T. (2003). Silent and apparent cerebral embolism after retrograde catheterisation of the aortic valve in valvular stenosis: a prospective, randomised study. Lancet.

[22] Redfors B., Watson B.M., McAndrew T., Palisaitis E., Francese D.P., Razavi M., Safirstein J., Mehran R., Kirtane A.J., Généreux P. (2017). Mortality, Length of Stay, and Cost Implications of Procedural Bleeding After Percutaneous Interventions Using Large-Bore Catheters. JAMA Cardiol..

