# Supplementary material for: Vascular complications and bleeding after balloon aortic valvuloplasty performed with or without heparin: HEPAVALVE randomized study
Source: Int J Cardiol Heart Vasc. 2022 Jan 18;39:100951. doi: 10.1016/j.ijcha.2021.100951 (PMC8984631; doi:10.1016/j.ijcha.2021.100951)
Supplement: Supplementary Table 1 [file mmc1.docx]

**Supplemental table 1. Endpoints assessment among patients receiving either heparin or placebo for balloon aortic valvuloplasty (Per-protocol analysis)**

|  | **All PP**  **N=77** | **UH group**  **n=37** | **Placebo group**  **n=40** | **_adj_OR**  **[95% CI]**  *UH vs. Placebo* | **p** |
| --- | --- | --- | --- | --- | --- |
| **Primary endpoint** | **7 (9.1)** | **6 (16.2)** | **1 (2.5)** | **10.8 [1.1-110.4]** | **0.04** |
| Major VC* | 4 (5.2) | 3 (8.1) | 1 (2.5) | 6.0 [0.5-73.9] | 0.2 |
| Major bleeding* | 1 (1.3) | 1 (2.7) | 0 (0.0) | 6.4 [0.5-84.3] | 0.2¤ |
| Major ischemic complication** | 3 (3.9) | 3 (8.1) | 0 (0.0) | 6.0 [0.5-66.6] | 0.1¤ |
| **Secondary endpoint** | **8 (10.4)** | **5 (13.5)** | **3 (7.5)** | **2.0 [0.3-14.0]** | **0.5** |
| Minor VC | 1 (1.3) | 0 (0.0) | 1 (2.5) | 0.6 [0.06-5.5] | 0.6¤ |
| Minor bleeding** | 7 (9.1) | 5 (13.5) | 2 (5.0) | 3.1 [0.4-26.5] | 0.3 |
| **Primary or secondary endpoint** | **14 (18.2)** | **10 (27.0)** | **4 (10.0)** | **4.1 [0.9-17.9]** | **0.06** |
| **Total vascular and bleeding** | **12 (15.6)** | **8 (21.6)** | **4 (10.0)** | **3.3 [0.7-16.12]** | **0.1** |

PP: per-protocol; VC: vascular complications

p_adj_ : p value of multivariate logistic regression with treatment group as covariate adjusted on diabetes, sex, coronary artery disease, renal failure and percutaneous closure device; ^¤^ p value estimated with Firth logistic regression.

*The same patient presented major VC and major bleeding.

**The same patient presented minor bleeding and major ischemic complication
