# Supplementary material for: Vascular complications and bleeding after balloon aortic valvuloplasty performed with or without heparin: HEPAVALVE randomized study
Source: Int J Cardiol Heart Vasc. 2022 Jan 18;39:100951. doi: 10.1016/j.ijcha.2021.100951 (PMC8984631; doi:10.1016/j.ijcha.2021.100951)
Supplement: Supplementary Table 2 [file mmc2.docx]

**Supplemental Table 2: Baseline and procedural characteristics of patients with and without major complications in intention-to-treat and per protocol analysis (primary endpoint).**

|  | **ITT population**  **n= 82** | | | **Per Protocol population**  **n= 77** | | |
| --- | --- | --- | --- | --- | --- | --- |
|  | **No complication**  **n= 75** | **Major complication**  **n=7** | **p-value** | **No complication**  **n=70** | **Major complication**  **n=7** | **p-value** |
| ***Patient characteristics*** | | | | | | |
| **Age (Years)*** | 85.0 (79.0-89.0) | 87.0 (84.0-89.0) | 0.3 | 84.5 (79.0-88.0) | 87.0 (84.0-89.0) | 0.3 |
| **Male Sex***, n(%)* | 34 (45.33) | 3 (42.9) | 1.0 | 31 (44.3) | 3 (42.9) | 1.0 |
| **BMI (km/m2)***** | 25.7 (23.3-29.7) | 23.5 (19.9-26.6) | 0.1 | 25.6 (23.3-29.7) | 23.5 (19.9-26.6) | 0.1 |
| **Hypertension***, n(%)* | 44 (59.5) | 6 (85.7) | 0.2 | 43 (62.3) | 6 (85.7) | 0.4 |
| **Coronary artery disease***, n(%)* | 28 (37.8) | 2 (28.6) | 1.0 | 26 (37.7) | 2 (28.6) | 1.0 |
| **Previous stroke***, n(%)* | 5 (6.8) | 2 (28.6) | 0.1 | 5 (7.3) | 2 (28.6) | 0.1 |
| **Permanent pacemaker***, n(%)* | 9 (12.0) | 1 (14.3) | 1.0 | 9 (12.9) | 1 (14.3) | 1.0 |
| **COPD***, n(%)* | 18 (24.0) | 4 (57.1) | 0.1 | 15 (21.4) | 4 (57.1) | 0.05 |
| **Atrial fibrillation***, n(%)* | 33 (45.2) | 4 (57.1) | 0.7 | 29 (42.7) | 4 (57.1) | 0.7 |
| **Diabetes***, n(%)* | 21 (28.4) | 1 (14.3) | 0.7 | 19 (27.5) | 1 (14.3) | 0.7 |
| **LVEF (%)*** | 44 (35-75) | 45 (40-75) | 0.3 | 43 (35-45) | 45 (40-75) | 0.3 |
| **Peripheral artery disease***,n(%)* | 10 (13.5) | 0 (0.0) | 0.3 | 10 (14.5) | 0 (0.00) | 0.3 |
| **NYHA ≥3***, n(%)* | 52 (75.4) | 6 (85.7) | 1.0 | 48 (75.0) | 6 (85.7) | 1.0 |
| **Renal failure***, n(%)* | 14 (18.7) | 2 (28.6) | 0.6 | 10 (14.3) | 2 (28.6) | 0.3 |
| **Hemoglobin <120 mmHg***, n(%)* | 35 (46.7) | 5 (71.4) | 0.3 | 32 (45.7) | 5 (71.4) | 0.3 |
| **Antithrombotic regimen***, n(%)* |  |  | 0.9 |  |  | 0.9 |
| None | 4 (10.3) | 5 (11.6) |  | 4 (10.8) | 5 (12.5) |  |
| SAPT | 14 (35.9) | 14 (32.6) |  | 14 (37.8) | 13 (32.5) |  |
| DAPT | 7 (17.9) | 7 (16.3) |  | 7 (18.9) | 7 (17.5) |  |
| Anticoagulant alone | 7 (17.9) | 10 (23.3) |  | 5 (13.5) | 9 (22.5) |  |
| Anticoagulant +SAPT | 5 (12.8) | 6 (13.9) |  | 5 (13.5) | 5 (12.5) |  |
| Anticoagulant +DAPT | 2 (5.1) | 1 (2.3) |  | 2 (5.4) | 1 (2.5) |  |
| ***Procedural characteristics*** | | | | | | |
| **Percutaneous closure device,** *n(%)* | 64 (87.7) | 7 (100.0) | 0.3 | 61 (87.1) | 7 (100.0) | 0.3 |
| **Femoral sheath size (French)***, n(%)* |  |  |  |  |  | 1.0 |
| *8 French* | 67 (93.1) | 7 (100.0) |  | 4 (5.7) | 0 (0.00) |  |
| *9 French* | 4 (5.6) | 0 (0.0) |  | 1 (1.4) | 0 (0.00) |  |
| **Number of inflations*** | 2.0 (2.0-3.0) | 2.0 (2.0-3.0) | 0.6 | 2.0 (2.0-3.0) | 2.0 (2.0-3.0) | 0.6 |
| **Balloon size (mm)*** | 22.0 (20.0-22.0) | 22.0 (20.0-22.0) | 0.3 | 22.0 (20.0-22.0) | 22.0 (20.0-22.0) | 0.2 |
| **Mean aortic gradient pre-BAV (mmHg)*** | 43.0 (32.0-56.0) | 40.0 (26.0-58.0) | 0.8 | 43.0 (32.0-56.0) | 40.0 (26.0-58.0) | 0.7 |
| **Mean aortic gradient post-BAV (mmHg)*** | 17.0 (9.0-29.0) | 17.5 (15.0-27.0) | 0.8 | 20.0 (10.0-29.0) | 17.5 (15.0-27.0) | 0.7 |

* Quantitative variables are expressed as median (Q_25_- Q_75_)

BAV: balloon aortic valvuloplasty; BMI: body mass index; COPD: chronic obstructive pulmonary disease; DAPT: dual antiplatelet therapy; GI: gastro-intestinal; ITT : intention-to-treat; LVEF: left ventricular ejection fraction; NYHA. New York Heart Association; PP: per-protocol; SAPT: simple antiplatelet therapy; UH: unfractionned heparin
