# Supplementary material for: Vascular complications and bleeding after balloon aortic valvuloplasty performed with or without heparin: HEPAVALVE randomized study
Source: Int J Cardiol Heart Vasc. 2022 Jan 18;39:100951. doi: 10.1016/j.ijcha.2021.100951 (PMC8984631; doi:10.1016/j.ijcha.2021.100951)
Supplement: Supplementary Table 3 [file mmc3.docx]

**Supplemental table 3. Antithrombotic regimen for each patient who had a major bleeding, major vascular complication, or major ischemic event.**

| **Patient number** | **Major complication** | **Antithrombotic regimen** |
| --- | --- | --- |
| 1 | Major bleeding complication + Major vascular complication | None |
| 2 | Major ischemic complication | SAPT |
| 3 | Major ischemic complication | Anticoagulant (stopped prior the BAV) |
| 4 | Major ischemic complication | SAPT + Anticoagulant (stopped prior the BAV) |
| 5 | Major vascular complication | SAPT + Anticoagulant (stopped prior the BAV) |
| 6 | Major vascular complication | SAPT + Anticoagulant (stopped prior the BAV) |
| 7 | Major vascular complication | DAPT |

BAV: balloon aortic valvuloplasty; DAPT: dual antiplatelet therapy; SAPT: simple antiplatelet therapy
